# Supplementary material for: Agent-Based Modelling of Health Inequalities following the Complexity Turn in Public Health: A Systematic Review
Source: Int J Environ Res Public Health. 2022 Dec 14;19(24):16807. doi: 10.3390/ijerph192416807 (PMC9779847; doi:10.3390/ijerph192416807)
Supplement: Supplementary file 1 [file ijerph-19-16807-s001.zip › ijerph-2074989-supplementary.pdf]

Table S1. Systematic Search Strategy.

Concept	Search Terms
Simulation Model	"agent based" OR "agent-based" OR "individual based" OR "individual-based" OR "multi agent*" OR "multi-agent*" OR "microsimulation*" OR "micro-simulation*" OR "simulation model*" OR "social simulation*"
Socioeconomic Inequality	injustice OR discrimination OR inequalit* OR disparit* OR equit* OR inequit* OR equalit* OR socioeconomic* OR socio-economic* OR "social determinant*" OR "social class*" OR "social grade*" OR sociodemographic* OR "social gradient*" OR disadvantage* OR unemploy* OR underemploy* OR poverty OR impoverished OR low-income OR "low income" OR occupation* OR education*
Health	sickness OR health OR healthy* OR healthi* OR illness* OR disabilit* OR morbidit* OR mortalit* OR disease OR diseases OR intervention*
